# M_4 _muscarinic receptor knockout mice display abnormal social behavior and decreased prepulse inhibition

**DOI:** 10.1186/1756-6606-5-10

**Published:** 2012-04-02

**Authors:** Hisatsugu Koshimizu, Lorene M Leiter, Tsuyoshi Miyakawa

**Affiliations:** 1 Division of Systems Medical Science, Institute for Comprehensive Medical Science, Fujita Health University, Toyoake 470-1192, Japan; 2 Core Research for Evolutional Science and Technology (CREST), Japan Science and Technology Agency, Kawaguchi 332-0012, Japan; 3 Howard Hughes Medical Institute, The Picower Center for Learning and Memory and RIKEN/Massachusetts Institute of Technology Neuroscience Research Center, Departments of Biology and Brain and Cognitive Sciences, Massachusetts Institute of Technology, Cambridge, MA 02139, USA

## Abstract

**Background:**

In the central nervous system (CNS), the muscarinic system plays key roles in learning and memory, as well as in the regulation of many sensory, motor, and autonomic processes, and is thought to be involved in the pathophysiology of several major diseases of the CNS, such as Alzheimer's disease, depression, and schizophrenia. Previous studies reveal that M_4 _muscarinic receptor knockout (M_4_R KO) mice displayed an increase in basal locomotor activity, an increase in sensitivity to the prepulse inhibition (PPI)-disrupting effect of psychotomimetics, and normal basal PPI. However, other behaviorally significant roles of M_4_R remain unclear.

**Results:**

In this study, to further investigate precise functional roles of M_4_R in the CNS, M_4_R KO mice were subjected to a battery of behavioral tests. M_4_R KO mice showed no significant impairments in nociception, neuromuscular strength, or motor coordination/learning. In open field, light/dark transition, and social interaction tests, consistent with previous studies, M_4_R KO mice displayed enhanced locomotor activity compared to their wild-type littermates. In the open field test, M_4_R KO mice exhibited novelty-induced locomotor hyperactivity. In the social interaction test, contacts between pairs of M_4_R KO mice lasted shorter than those of wild-type mice. In the sensorimotor gating test, M_4_R KO mice showed a decrease in PPI, whereas in the startle response test, in contrast to a previous study, M_4_R KO mice demonstrated normal startle response. M_4_R KO mice also displayed normal performance in the Morris water maze test.

**Conclusions:**

These findings indicate that M_4_R is involved in regulation of locomotor activity, social behavior, and sensorimotor gating in mice. Together with decreased PPI, abnormal social behavior, which was newly identified in the present study, may represent a behavioral abnormality related to psychiatric disorders including schizophrenia.

## Background

Members of the muscarinic acetylcholine receptor family (M_1_-M_5_R) are widely expressed in the central nervous system (CNS) and the peripheral nervous system (PNS) [1,2]. CNS muscarinic receptors play key roles in learning and memory, as well as in the regulation of many sensory, motor, and autonomic processes, while PNS muscarinic receptors mediate the activity of acetylcholine released from parasympathetic nerves [3]. Reduced or increased signaling through distinct muscarinic acetylcholine receptor subtypes is implicated in the pathophysiology of several major diseases of the CNS, including Alzheimer's and Parkinson's diseases, depression, epilepsy, and schizophrenia [3-7].

Based on the overlapping expression patterns of the different muscarinic receptor subtypes [8-11] and the lack of ligands that display a high degree of receptor subtype selectivity, determining the precise functional roles of the individual muscarinic receptor species has been difficult [3,12,13]. To elucidate the roles of the individual M_1_-M_5_R, gene targeting technology has been used to produce mutant mouse lines containing inactivating mutations of the M_1_-M_5_R genes, and these knockout (KO) mice have been evaluated in a battery of physiological, pharmacological, biochemical, neurochemical, and behavioral tests [14].

The M_2_R and M_4_R are both linked to G proteins of the Gi family and share similar ligand binding properties, which makes it difficult to distinguish between these two receptor subtypes by classical pharmacological tools [3,12,13]. Previous studies with M_2_R and M_4_R KO mice reveal that M_2_R and M_4_R have several physiological and pharmacological functions in common. M_2_R is widely expressed in the CNS and in the body periphery, particularly in the heart and smooth muscle tissues [3,8,10,12]. By contrast, M_4_R is preferentially expressed in the CNS, particularly in different areas of the forebrain [8-10]. M_4_R is also expressed abundantly in the striatum and is present at lower levels in several other brain regions including the cerebral cortex and hippocampus [11,15].

Previous studies reveal that M_4_R is involved in locomotor activity, sensorimotor gating, and learning and memory in rodents. Gomeza et al. reported that M_4_R KO mice displayed a small but statistically significant increase in basal locomotor activity and a hypersensitivity to the stimulatory locomotor effects of D1R activation [16]. M_4_R is preferentially expressed by striatal projection neurons that express D1R in the striatum [17-19], and direct striato-nigral pathway activation is predicted to facilitate locomotion [17]. Felder et al. reported that male M_4_R KO mice exhibited normal startle response and a significant increase in sensitivity to the prepulse inhibition (PPI)-disrupting effect of a noncompetitive NMDA receptor antagonist, phencyclidine, whereas no significant difference in basal PPI was observed between genotypes [20]. A recent study reported that female M_4_R KO mice showed significantly increased startle response and normal PPI [21].

We previously assessed the role of M_2_R in learning and memory, demonstrating that M_2_R KO mice showed significant deficits in behavioral flexibility and working memory in the Barnes circular maze and T-maze delayed alternation tests, respectively [22]. By contrast, only a few studies to date have assessed the cognitive function of M_4_R in mice. Tazavara et al. reported that M_2_R KO and M_2_R/M_4_R KO, but not M_4_R KO, mice demonstrated impaired memory retention in a passive avoidance test [23]. Degroot and Nomikos reported that in the shock-probe burying model, M_4_R KO, but not M_2_R KO, mice exhibited significantly decreased burying behavior and normal long-term memory performance [24]. In rats, administration of muscarinic toxin 3, which has high selectivity for M_4_R and low affinity to other muscarinic receptors, enhanced memory retrieval in an inhibitory avoidance test [25]. M_4_R was also reported not to play a pronounced role in mediating muscarinic receptor-dependent analgesia, tremor, hypothermia, or salivation [16]. However, other behaviorally significant roles of M_4_R remain unaddressed, such as whether M_4_R is involved in social behavior, and spatial reference, working, and episodic-like memory.

In the present study, to further clarify the roles of M_4_R in the CNS, we subjected M_4_R KO mice to a battery of behavioral tests, including hot plate, wire hang, rotarod, open field, light/dark transition, social interaction, startle response/PPI, and reference and working memory version/delayed matching-to-place (DMP) tasks of the Morris water maze test, revealing that M_4_R deficiency led to increased novelty-induced locomotor activity, abnormal social behavior, and decreased PPI, while it did not affect spatial reference, working, or episodic-like memory in mice.

## Results

### M_4_R KO mice exhibited normal nociception and motor abilities

**Figure 1:**
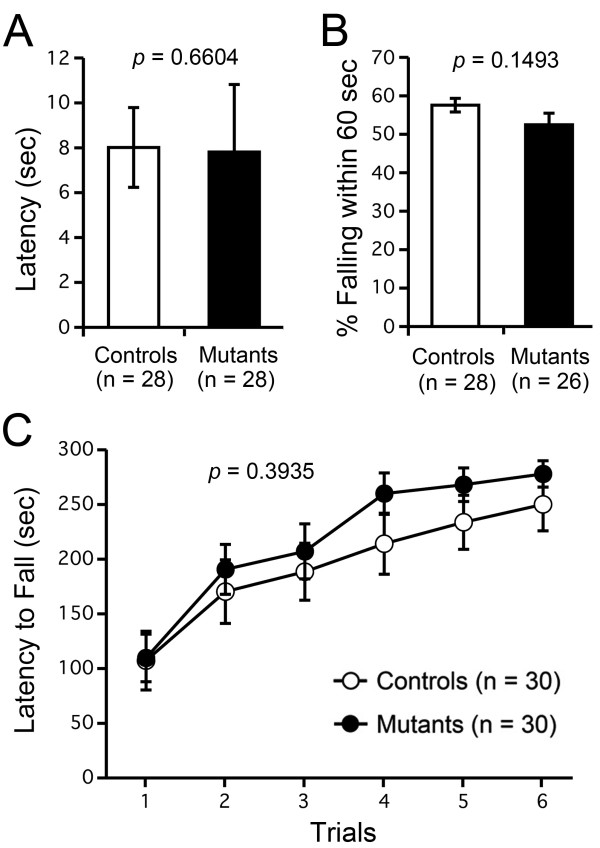
**Normal nociception and motor function of M_4_R KO mice**. (A) No difference in latency was observed between genotypes in the hot plate test. M_4_R KO mice (Mutants), n = 28; wild-type mice (Controls), n = 28. (B) No significant difference in percent falling within 60 s was observed between genotypes in the wire hang test. M_4_R KO mice, n = 26; wild-type mice, n = 28. (C) No significant differences in latency to fall were observed between genotypes in the rotarod test. M_4_R KO mice, n = 30; wild-type mice, n = 30.

M_4_R KO mice and their wild-type littermates were subjected to hot plate, wire hang, and rotarod tests. In the hot plate test, the latencies for M_4_R KO mice and wild-type mice were 8.014 ± 0.306 s and 7.812 ± 0.344 s, respectively, with no significant difference observed between genotypes (F_1,52 _= 0.195, *P *= 0.6604; Figure 1A). In the wire hang test, no significant difference in percent falling within 60 s was detected between genotypes (F_1,54 _= 2.140, *P *= 0.1493; Figure 1B). In the rotarod test, there was also no significant difference between genotypes in latency to fall (F_1,28 _= 0.751, *P *= 0.3935; Figure 1C). Thus, these tests failed to detect abnormalities in nociception, neuromuscular strength, or motor coordination/learning in M_4_R KO mice.

### M_4_R KO mice showed locomotor hyperactivity in open field and light/dark transition tests

**Figure 2:**
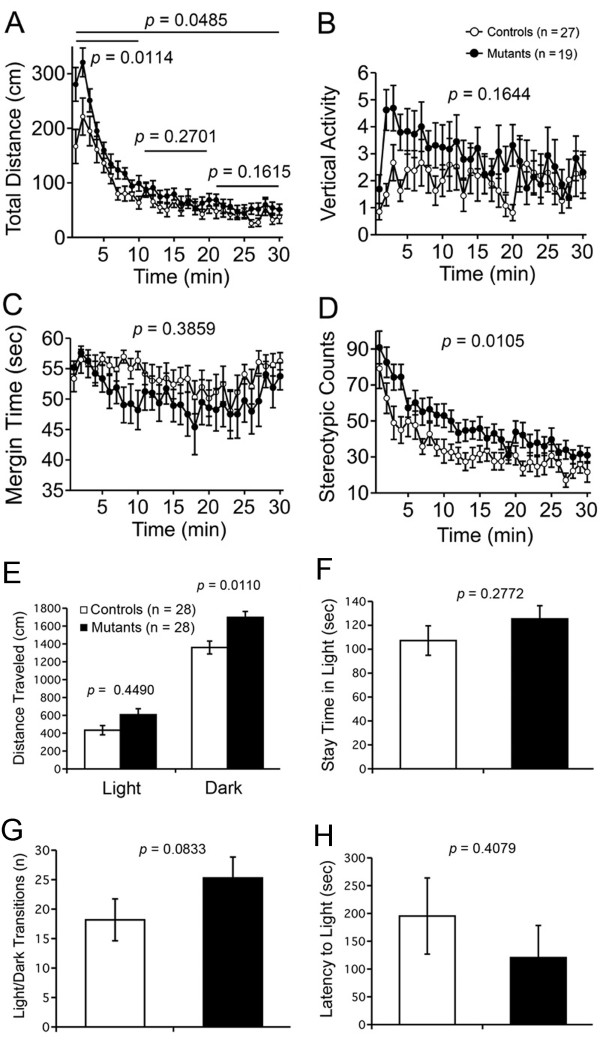
**Locomotor hyperactivity of M_4_R KO mice**. (A-D) M_4_R KO mice displayed an increase in novelty-induced locomotor hyperactivity in the open field test. (A) M_4_R KO mice traveled significantly longer than wild-type mice during the whole experimental period. Locomotor hyperactivity of M_4_R KO was detected in the earlier (1-10 min) part of the experiment but not in the middle (11-20 min) or later (21-30 min) parts of the experimental period. No significant differences were observed between genotypes in vertical activity (B) or margin time (C). (D) M_4_R KO mice demonstrated a significant increase in stereotypic behavior counts. M_4_R KO, n = 19; wild-type mice, n = 27. (E-H) M_4_R KO mice exhibited locomotor hyperactivity in the light/dark transition test. (E) M_4_R KO mice traveled significantly longer than wild-type mice in the dark box but not in the light box. No significant difference was observed between genotypes in time spent in the light box (F), the number of transitions between the light and dark sides (G), or latency to entering the light box (H). M_4_R KO mice, n = 28; wild-type mice, n = 28.

We next subjected M_4_R KO mice to the open field test. M_4_R KO mice traveled longer distances compared to wild-type mice over the entire experimental period (genotype effect, F_1,54 _= 4.075, *P *= 0.0485; genotype × time, F_1,29 _= 3.240, *P *< 0.0001). Locomotor hyperactivity was detected in M_4_R KO mice during the earlier part of the experimental period (1-10 min, F_1,54 _= 6.862, *P *= 0.0114), but not during the middle or later parts of the experimental period (11-20 min, F_1,54 _= 1.241, *P *= 0.2701; 21-30 min, F_1,54 _= 2.015, *P *= 0.1615), indicating novelty-induced locomotor hyperactivity in M_4_R KO mice (Figure 2A). No differences in vertical activity (F_1,54 _= 1.987, *P *= 0.1644; Figure 2B) or margin time (F_1,28 _= 0.776, *P *= 0.3859; Figure 2C) were observed between genotypes. M_4_R KO mice displayed a significant increase in stereotypic behavior counts (F_1,54 _= 7.022, *P *= 0.0105; Figure 2D).

In the light/dark transition test, M_4_R KO mice traveled significantly longer than wild-type mice (F_1,54 _= 11.905, *P *= 0.0110) in the dark box, indicating locomotor hyperactivity of M_4_R KO mice, while no significant difference in locomotor activity was seen in the light box (F_1,54 _= 4.215, *P *= 0.4490; Figure 2E). Time spent in the light box (F_1,54 _= 1.205, *P *= 0.2772; Figure 2F), the number of transitions between the light and dark sides (F_1,54 _= 3.113, *P *= 0.0833; Figure 2G), and latency to enter the light box (F_1,54 _= 0.696, *P *= 0.4079; Figure 2H) did not significantly differ between genotypes. These findings in the open field test and light/dark transition test confirmed previous studies revealing that lack of M_4_R led to locomotor hyperactivity in mice [16,26].

### M_4_R KO mice displayed abnormal social behavior in social interaction test

**Figure 3:**
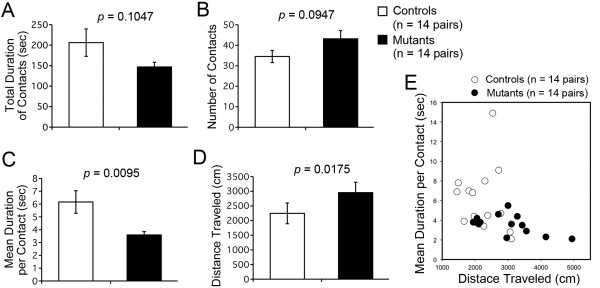
**Abnormal social behavior of M_4_R KO mice**. (A) No significant difference in total duration of contacts was detected between genotypes. (B) The number of contacts was slightly but not significantly increased in M_4_R KO mice. (C) M_4_R KO mice displayed a significant decrease in the mean duration of each contact compared to wild-type mice. (D) The distance traveled by M_4_R KO mice was significantly increased compared to that of wild-type mice. (E) Scatter plot of distance traveled against mean duration per contact. M_4_R KO mice, n = 14 pairs; wild-type mice, n = 14 pairs.

M_4_R KO mice were then subjected to the social interaction test. Neither the total duration of contacts (F_1,26 _= 2.827, *P *= 0.1047; Figure 3A) nor the number of contacts (F_1,26 _= 3.008, *P *= 0.0947; Figure 3B) significantly differed between genotypes. M_4_R KO mice displayed a highly significant decrease (F_1,26 _= 7.846, *P *= 0.0095) in mean duration per contact compared to wild-type mice (Figure 3C), and M_4_R KO mice also traveled significantly longer than wild-type mice (F_1,26 _= 6.445, *P *= 0.0175; Figure 3D). Because increased locomotor activity may increase the possibility of accidental contacts between mice, which could potentially cause a decrease in average contact time, we next assessed whether the increased locomotor activity of M_4_R KO mice accounted for their decreased mean duration per contact. A scatter plot of distance traveled against mean duration per contact indicated that even in M_4_R KO mice that did not show hyperactivity, mean duration per contact tended to be shorter than that of wild-type mice (Figure 3E). This difference, however, may have been due to the small sample size of wild-type mice (n = 14 pairs).

To address whether a decrease in mean duration per contact in M_4_R KO mice is a typical behavioral phenotype of hyperactive mutant mice, we next compared M_4_R KO mice with several gene-targeted mouse lines that exhibit hyperactive phenotypes, including calcineurin (CN) KO [27], M_1_R KO [28], alpha-isoform of calcium/calmodulin-dependent protein kinase II (αCaMK II) heterozygous KO [29], and neuronal nitric oxide synthase (nNOS) KO mice [30]. Among them, only CN KO mice, which display multiple abnormal behaviors related to schizophrenia, showed a decrease in mean duration per contact and an increase in distance traveled (Additional file 1: Table S1) [27]. In the remaining hyperactive gene-targeted mouse lines, the mean duration per contact was normal compared to that of wild-type littermates, while locomotor activity was significantly increased (Additional file 1: Table S1) [28-30], suggesting that a decrease in mean duration per contact of M_4_R KO mice is not a typical behavioral phenotype for hyperactive mice and is not likely caused by their hyperactivity. These findings indicate that lack of M_4_R caused abnormal social behavior in mice.

### M_4_R KO mice exhibited normal startle response and decreased PPI

**Figure 4:**
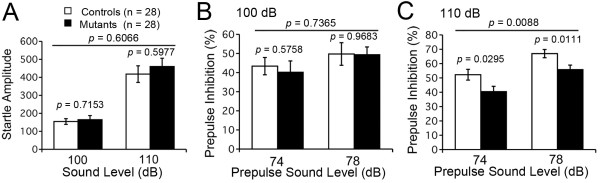
**Normal startle response and decreased PPI in M_4_R KO mice**. (A) M_4_R KO mice demonstrated normal acoustic startle response for the 100 and 110 dB startle stimulus. (B) No significant differences in PPI for the 74 and 78 dB prepulse sound level followed by 100 dB startle stimulus were observed between genotypes. (C) M_4_R KO mice demonstrated a significant decrease in prepulse inhibition for the 74 and 78 dB prepulse sound level followed by 110 dB startle stimulus compared to wild-type mice. M_4_R KO mice, n = 28; wild-type mice, n = 28.

Next, M_4_R KO mice were subjected to startle response/PPI tests. M_4_R KO mice demonstrated normal acoustic startle response for the 100 dB and 110 dB startle stimulus (100 and 110 dB, F_1, 54 _= 0.268, *P *= 0.6066; 100 dB, F_1, 54 _= 0.134, *P *= 0.7153; 110 dB, F_1, 54 _= 0.282, *P *= 0.5977; Figure 4A). PPI did not significantly differ from wild-type mice for the 74 and 78 dB prepulse sound levels followed by 100 dB startle stimulus (74 and 78 dB, F_1, 54 _= 0.114, *P *= 0.7365; 74 dB, F_1, 54 _= 0.317, *P *= 0.5758; 74 and 78 dB, F_1, 54 _= 0.003, *P *= 0.9683; Figure 4B). By contrast, M_4_R KO mice demonstrated a significant decrease in PPI for the 74 and 78 dB prepulse sound level followed by 110 dB startle stimulus compared to wild-type mice (74 and 78 dB, F_1, 54 _= 7.401, *P *= 0.0088; 74 dB, F_1, 54 _= 4.998, *P *= 0.0295; 78 dB, F_1, 54 _= 6.918, *P *= 0.0111; Figure 4C), indicating that M_4_R KO mice exhibit impaired PPI when the intensity of startle stimulus is high.

### M_4_R KO mice displayed normal spatial reference, working, and episodic-like memory in Morris water maze test

**Figure 5:**
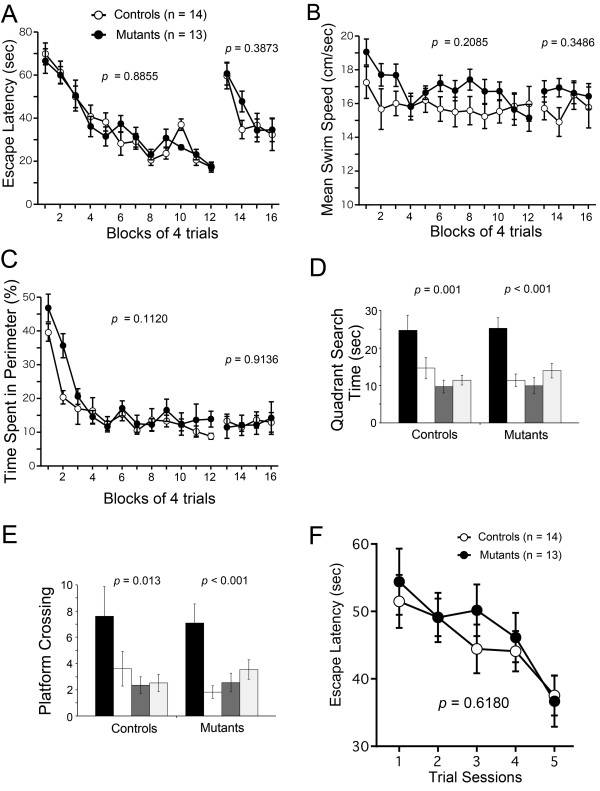
**Normal spatial reference, working, and episodic-like memory of M_4_R KO mice in the Morris water maze**. (A-E) No significant differences in the conventional hidden platform version of the Morris water maze were detected between genotypes. Escape latency (A), swimming speed (B), and time spent in the perimeter of the pool (C) did not differ significantly between the two genotypes during either original or reversal learning. M_4_R KO mice, n = 13; wild-type mice, n = 14. In probe trials, both M_4_R KO mice and wild-type mice selectively searched the location where the platform had been located. M_4_R KO mice, n = 11; wild-type mice, n = 12. (D) Both genotypes spent significantly more time in the training quadrant (black bars) compared with the other quadrants (opposite quadrant, white bars; right quadrant, dark gray bars; left quadrant, light gray bars) in the probe trials conducted after original training. (E) Also, both genotypes crossed the training site significantly more often than the equivalent sites in the other three quadrants in the probe trials. (F) No significant difference in the delayed matching-to-place (DMP) task of the Morris water maze was detected between genotypes. M_4_R KO mice showed normal latency in the first five trials of new platform training, averaged over six training sessions. M_4_R KO mice n = 13; wild-type mice, n = 14.

Finally, M_4_R KO mice were subjected to the conventional hidden platform version and DMP task of the Morris water maze test to evaluate spatial reference, working, and episodic-like memory in mice. No significant differences between genotypes were observed in latency to platform (time required to reach the platform) during original learning (F_1, 25 _= 0.021, *P *= 0.8855) or reversal learning (F_1, 25 _= 0.774, *P *= 0.3873; Figure 5A), swimming speed during original learning (F_1, 25 _= 1.667, *P *= 0.2085) or reversal learning (F_1, 25 _= 0.912, *P *= 0.3486; Figure 5B), or time spent at the perimeter of the pool during original learning (F_1, 25 _= 2.713, *P *= 0.1120) or reversal learning (F_1, 25 _= 0.012, *P *= 0.9136; Figure 5C). During probe trials, in which the platform was removed, both M_4_R KO mice and wild-type mice selectively searched for the location where the platform had been located. Both genotypes spent significantly more time in the training quadrant compared with the other quadrants (M_4_R KO mice, F_3, 40 _= 6.52905, *P *< 0.001; wild-type mice, F_3, 36 _= 16.03608, *P *= 0.001) in the probe trials conducted after original training (Figure 5D). Also, both genotypes crossed the training site significantly more often than the equivalent sites in the other three quadrants (M_4_R KO mice, F_3, 40 _= 4.06293, *P *< 0.001; wild-type mice, F_3, 36 _= 8.85831, *P *= 0.013) in the probe trials (Figure 5E). In the DMP task, no significant difference in latency to platform was observed between genotypes (*P *= 0.6180; Figure 5F). These data indicate that lack of M_4_R does not induce impairments in spatial reference, working, or episodic-like memory in mice.

## Discussion

In the present study, to clarify the roles of muscarinic acetylcholine receptor M_4 _in the CNS, we performed a behavioral test battery on M_4_R KO mice and identified physical and behavioral phenotypes, including locomotor hyperactivity, abnormal social behavior, and decreased PPI. These abnormal behaviors in mice are thought to be correlates of the symptoms of schizophrenia, including locomotor hyperactivity, abnormal social behavior, and sensorimotor gating deficits [31-33].

In light/dark transition test, M_4_R KO mice traveled longer distances than wild-type mice in the dark box, while no significant differences between genotypes were detected in distance traveled, time spent in the light box, the number of transitions between the light and dark sides, or latency to enter the light box. These observations indicate that M_4_R is involved in modulation of locomotor activity in mice. This finding is consistent with previous studies, in which M_4_R KO mice demonstrated an increase in basal locomotor activity and greatly enhanced locomotor responses with activation of D1R [16,26]. In the open field test, M_4_R KO mice traveled longer than their wild-type littermates, but the difference between genotypes in distance traveled was observed only in the earlier part of the test, not in the middle or later parts of the test. It is likely that reactivity to novelty was enhanced in M_4_R KO mice, leading to novelty-induced locomotor hyperactivity.

To our knowledge, this study is the first to evaluate the social behavior of M_4_R KO mice. No significant differences between M_4_R KO mice and wild-type control mice were observed in total duration of contacts between pairs, while average duration of each contact between pairs of M_4_R KO mice was significantly shorter than that of wild-type mice. Because M_4_R KO mice traveled longer than wild-type mice, we assessed whether their increased locomotor activity underlies their decreased average duration of each contact using other gene-targeted mouse lines exhibiting locomotor hyperactivity, including M_1_R KO, αCaMK II heterozygous KO, nNOS KO, and CN KO mice. Among these hyperactive mice, decreased contact time was only observed in CN KO mice, which exhibit multiple abnormal behaviors related to schizophrenia (Additional file 1: Table S1) [28-30], indicating that M_4_R KO and CN KO mice have decreased exploration compared to other mice, while most hyperactive mice maintain exploratory behavior toward other mice.

In the present study, we observed that M_4_R KO mice displayed a significant decrease in PPI, whereas M_4_R KO mice exhibited normal basal PPI in a previous study reported by Felder et al. [20]. This discrepancy might be due to differences in the experimental conditions: in the previous study, the intensities of startle and prepulse stimuli were higher than those used in the present study. Additionally, other differences in experimental conditions, such as the age of animals or apparatus configuration, may account for the discrepancy. It is also possible that experiences and/or stress during the behavioral test battery carried out prior to the startle response/PPI tests enhanced the genotype effect on sensorimotor gating in M_4_R KO mice.

In addition to a decrease in PPI, M_4_R KO mice also exhibited hyperactivity and abnormal social behavior. Notably, those behavioral abnormalities were also observed in other schizophrenia model mice, such as CN KO mice [27] and AMPA GluR1 receptor KO mice [33]. Accumulating evidence indicates that M_4_R and M_2_R are involved in schizophrenia and related psychiatric disorders. Post-mortem CNS studies report a significant decrease in expression levels of M_4_R and M_2_R in schizophrenia [34,35]. In addition, neuropsychopharmacological studies suggest that the antipsychotic clozapine, which is used in the treatment of schizophrenia, is a partial agonist of muscarinic receptors including M_4_R and M_2_R [35,36]. Xanomeline, which has been assessed for the treatment of schizophrenia, is an M_4_R and M_1_R agonist [35,37]. Thus, M_4_R and M_2_R in the CNS are thought to be potential drug targets for the treatment of schizophrenia and related neurological disorders [3].

Muscarinic receptor involvement in the CNS has been indicated in spatial learning and encoding of new episodic memory based on various lines of evidence. Muscarinic antagonists, such as scopolamine [38,39] and atropine [39-41], induce impairments in cognitive performance in rodents. Scopolamine and the cholinotoxin IgG 192-saporin induce blockage of long-term potentiation enhancement in rats [42]. Theta rhythm oscillations, which are thought to function in encoding new episodic memory in the hippocampal formation, are reduced by atropine in rats [39,43]. In the present study, we assessed whether M_4_R is involved in spatial reference, working, and episodic-like memory in the Morris water maze test and revealed no differences between genotypes. These findings demonstrated that the lack of M_4_R does not lead to significant cognitive deficits in spatial reference, working, and episodic-like memory in mice. Given that knockout of M_2_R induces behavioral flexibility, working memory, and hippocampal plasticity in mice [22], it is possible that functional compensation by M_2_R accounts for the normal cognitive performance of M_4_R KO mice in these tests.

## Conclusion

In summary, our battery of behavioral tests on M_4_R KO mice indicate that M_4_R plays a role in modulating locomotor activity, social behavior, and sensory motor gating. Further studies are required to elucidate the precise molecular mechanisms by which M_4_R regulates behavioral phenotypes and to address how M_4_R is involved in psychiatric disorder-related behavioral abnormalities.

## Methods

### Animals and experimental design

M_4_R KO mice were generated in 129SvEv embryonic stem (ES) cells and maintained in a pure 129SvEv background (kindly provided by Dr. Jürgen Wess, National Institute of Diabetes and Digestive and Kidney Disease, National Institutes of Health, Bethesda, MD). All behavioral tests were carried out with male mice (Table 1). Mice were housed in a room with a 12-h light/dark cycle (lights on at 7:00 a.m.) and access to food and water *ad libitum*. Behavioral testing was performed between 9:00 a.m. and 5:00 p.m. All procedures relating to animal care and treatment conformed to Massachusetts Institute of Technology and National Institutes of Health guidelines.

**Table 1:** Behavioral test battery of M_4_R KO mice

Test	Age	Results
Open Field	8-13 w	Figure 2
L/D Transition	9-13 w	Figure 2
Hot Plate	9-14 w	Figure 1A
Rotarod	10-14 w	Figure 1C
Wire Hang	10-15 w	Figure 1B
PPI	11-15 w	Figure 4
Social Interaction	12-16 w	Figure 3
Reference and Working Memory Versions of the Morris Water Maze	21-28 w	Figure 5A-E
DMP Task of the Morris Water Maze	24-29 w	Figure 5F

### Hot plate test, wire hang test, and rotarod test

The hot plate test was used to evaluate sensitivity to a painful stimulus. Mice were placed on a 55.0°C (± 0.3°C) hot plate (Columbus Instruments, Columbus, OH), and latency to the first hind paw response was recorded. The hind paw response was either a foot shake or a paw lick. In the wire hang test, the mouse was placed on a wire mesh that was then inverted and waved gently, so that the mouse gripped the wire. Latency to fall was recorded with a 60-s cut-off time. The rotarod test was performed using an accelerating rotarod (Accelerating Rotarod, UGO Basile, Collegeville, PA) and consisted of placing a mouse on a rotating drum (3-cm diameter) and measuring the time each animal was able to maintain its balance on the rod. The speed of the rotarod accelerated from 4 to 40 rpm over a 5-min period.

### Open field test

Each mouse was placed in the center of an open field apparatus (40 × 40 × 30 cm; Accuscan Instruments, Columbus, OH). Total distance traveled (in cm), vertical activity, time spent in close proximity (within 1 cm) to the wall of the cage, and the beam-break counts for stereotyped behaviors were recorded by IMAGE OF software (O'Hara & Co, Tokyo, Japan). Data were collected over a 30-min period.

### Light/dark transition test

The light/dark transition test was used to measure anxiety-like behavior [44]. The apparatus used for the light/dark transition test consisted of a cage (21 × 42 × 25 cm) divided into two sections of equal size by a black partition containing a small opening (O'Hara & Co.). One chamber was brightly illuminated, whereas the other chamber was dark. Mice were placed into the illuminated side and allowed to move freely between the two chambers for 10 min. The total number of transitions, time spent in the dark side, and distance traveled were recorded by IMAGE LD4 software (O'Hara & Co).

### Social interaction test

The social interaction test in a novel environment was performed in a manner similar to published methods [27] to measure social behavior in mice. Two mice of identical genotypes, which were previously housed in different cages, were placed into a box together (40 × 40 × 30 cm) and allowed to explore freely for 10 min. Social behavior was monitored by a CCD camera (DXC-151A, Sony, Tokyo, Japan) connected to a Macintosh computer. Analysis was performed automatically using IMAGE SI software (O'Hara & Co). The number of contacts, mean duration per contact, and total distance traveled were measured.

### Startle response/prepulse inhibition tests

A startle reflex measurement system (MED Associates, St. Albans, VT) was used to measure startle response and PPI. A test session began by placing a mouse in a Plexiglas cylinder, where it was left undisturbed for 5 min. The duration of white noise used as the startle stimulus was 40 ms for all trial types. The startle response was recorded for 160 ms (measuring the response every 1 ms) starting with the onset of the prepulse stimulus. The background noise level in each chamber was 70 dB. The peak startle amplitude recorded during the 160-ms sampling window was used as the dependent variable. A test session consisted of six trial types (i.e., two types for startle stimulus only trials, and four types for prepulse inhibition trials). The startle stimulus intensity was 100 or 110 dB. The prepulse sound was presented 100 ms before the startle stimulus, and its intensity was 74 or 78 dB. Four combinations of prepulse and startle stimuli were employed (74-100, 78-100, 74-110, and 78-110 dB). Six blocks of the six trial types (four trial types with the combinations of prepulse and startle stimulus and two startle stimulus only trials) were presented in pseudorandom order such that each trial type was presented once within a block. The average inter-trial interval was 15 s (range: 10-20 s).

### Morris water maze test

The conventional hidden platform version of the Morris water maze test was used to test spatial reference and working memory in mice. The pool was 160 cm in diameter and made opaque by covering the water surface with tiny resin beads (Hanna Resin Distribution, MA). Water temperature was maintained at room temperature (19°C). The platform was 15 cm in diameter. Each mouse was trained in four trials per day with inter-trial intervals of 30-60 min. In each trial, the mouse was allowed to swim until it found the platform or until 90 s had elapsed, at which point the mouse was guided to the platform. The mouse was then allowed to sit on the platform for 30 s before it was picked up. The duration of each probe test was 60 s. No mouse was exposed to any pretraining in this reference memory task. All experiments were videotaped, and the tapes were later digitized and analyzed using Image WM software (O'Hara & Co.). The DMP task of the Morris water maze test was conducted as described [45] with minor modifications to evaluate working and episodic-like memory in mice. The mice were first pretrained to a visible platform for 3 days, with four trials per day and inter-trial intervals of 20-40 min. The mice were then trained repeatedly to navigate to a hidden platform at a fixed location until reaching a rigorous criterion of three consecutive trials with an average escape latency of less than 20 s or until completing a maximum of 24 trials. Each mouse performed up to eight trials per day with inter-trial intervals of 10 min. If a mouse reached the criterion in fewer than five trials, it was continually trained to complete five trials, so that a complete set of latency data from all mice could be obtained for the first five trials. After the training was completed (which took up to 3 days), starting the following day, the mice were trained to a new hidden platform location in the same manner as the training to the first location except that the maximal number of trials was reduced to 16. This protocol was repeated four more times until a total of six platform locations were learned. All water maze experiments, including this DMP task and the above reference memory task, were videotaped, and the tapes were later digitized and analyzed with Image WM software (O'Hara & Co).

### Image analysis

All applications used for the behavioral studies (Image OF, Image LD4, Image SI, and Image WM) were run on Macintosh computers. Applications were based on the public domain NIH IMAGE program (developed by Dr. Wayne Rasband at the National Institute of Mental Health, Bethesda) and were modified for each test by Dr. Tsuyoshi Miyakawa [28,46] (available through O'Hara & Co.).

### Statistical analysis

Statistical analysis was conducted by using Statview (SAS Institute, Cary, NC). Data were analyzed by one-way or two-way analysis of variance (ANOVA), or analysis of covariance (ANCOVA). Post hoc analyses were performed on all ANOVAs found to be significant. Values in graphs are expressed as mean ± SEM.

## Abbreviations

M_1_-M_5_R: Muscarinic acetylcholine receptor 1-5; CNS: Central nervous system; PNS: Peripheral nervous system; KO: Knockout; PPI: Prepulse inhibition; αCaMK II heterozygous KO: Alpha-isoform of calcium/calmodulin-dependent protein kinase II heterozygous knockout; nNOS: Neuronal nitric oxide synthase; CN: Calcineurin.

## Competing interests

The authors declare that they have no competing interests.

## Authors' contributions

Conceived and designed the experiments: TM. Performed the experiments: LML TM. Analyzed the data: HK MT. Wrote the paper: HK MT. All authors read and approved the final manuscript.

## Supplementary Material

Additional file 1 **Table S1 Number of contacts, mean duration per contact, and distance traveled in M_4_R KO, M_1_R KO, αCaMK II heterozygous KO, nNOS KO, and CN KO mice**. Click here for file
