# Supplementary material for: M4 muscarinic receptor knockout mice display abnormal social behavior and decreased prepulse inhibition
Source: Mol Brain. 2012 Apr 2;5:10. doi: 10.1186/1756-6606-5-10 (PMC3361477; doi:10.1186/1756-6606-5-10)
Supplement: Additional file 1 — Table S1 Number of contacts, mean duration per contact, and distance traveled in M4R KO, M1R KO, αCaMK II heterozygous KO, nNOS KO, and CN KO mice. [file 1756-6606-5-10-S1.DOCX]

| **Supplemental Table 1** | | |  | |  |
| --- | --- | --- | --- | --- | --- |
| Number of contacts, mean duration per contact, and distance traveled in M_4_R KO, M_1_R KO, αCaMK II heterozygous KO, nNOS KO, and CN KO mice | | | | | |
|  | Number of  Contacts | Mean Duration  per Contact (s) | | Distance  Traveled (cm) | |
| M_4_R KO  WT | 43.1 ± 4.0  34.5 ± 3.0  *P* = 0.0947 | 3.6 ± 0.3  6.2 ± 0.9  *P* = 0.0095 | | 2954.7 ± 237.4  2246.3 ± 146.6  *P* = 0.0175 | |
| M_1_R KO  WT | 26.6 ± 2.1  17.8 ± 1.8  *P* = 0.0032 | 3.6 ± 0.2  3.9 ± 0.3  *P* = 0.4233 | | 2803.8 ± 233.6  1884.2 ± 147.3  *P* = 0.0029 | |
| αCaMK II heterozygous KO  WT | 68.5 ± 4.3  61.3 ± 3.4  *P* > 0.05 | 0.9 ± 0.1  1.0 ± 0.1  *P* > 0.05 | | 5851.1 ± 269.0  4436.8 ± 293.0  *P* = 0.0032 | |
| nNOS KO  WT | 74.0 ± 3.5  62.1 ± 4.4  *P* = 0.0456 | 1.5 ± 0.1  1.6 ± 0.1  *P* = 0.7507 | | 3980.8 ± 195.3  3125.4 ± 147.7  *P* = 0.0017 | |
| CN KO  WT | 7.4 ± 0.3  6.9 ± 0.1  *P* = 0.2400 | 1.0 ± 0.1  1.5 ± 0.1  *P* < 0.0001 | | 753.4 ± 78.0  586.2 ± 38.8  *P* = 0.0316 | |

Red and blue indicate increase and decrease in mutants, respectively.

M_4_R KO: M_4_ muscarinic receptor knockout; M_1_R: M_1_ muscarinic receptor; αCaMK II heterozygous KO: alpha-isoform of calcium/calmodulin-dependent protein kinase II heterozygous knockout; nNOS: neuronal nitric oxide synthase; CN: calcineurin; WT: wild type.

Genetic backgrounds: M4R KO, 129 SvEv; M1R KO, C57BL/6J × 129 SvEv hybrids (F2 littermates); αCaMKII heterozygous KO, C57BL/6J; nNOS KO, B6;129S4-Nos1^tm1hlh^/J backcrossed for five generations onto a C57BL/6J background; CN KO, C57BL/6J
